# Antioxidant Phenolic Substances of Turkish Red Wines from Different Wine Regions

**DOI:** 10.3390/molecules14010289

**Published:** 2009-01-09

**Authors:** R. Ertan Anli, Nilüfer Vural

**Affiliations:** 1 Department of Food Engineering, Faculty of Engineering, Ankara University, 06110 Diskapi, Ankara, Turkey; 2 Ankara University Biotechnology Institute, Ankara, Turkey; E-mail: nvural@science.ankara.edu.tr (N. V.)

**Keywords:** HPLC, Antioxidant phenolics, Red wines

## Abstract

In this study, five biologically phenolic antioxidant Turkish red wines from different regions of Turkey were determined using HPLC with PDA detection. The antioxidant capacities (AC) of the investigated wines are also determined and the relationship between the phenol content and antioxidant capacity is discussed. The results show that Kalecik Karası, Merlot and Cabernet Sauvignon AC values ranged between 15.8-18.7 mmol/L, 15.8-17.6 mmol/L and 18.1-22.6 mmol/L, respectively. Generally, Cabernet Sauvignon wines not only had higher levels of phenolic antioxidants, such as catechin, epicatechin and gallic acid, but also higher antioxidant capacities compared to Kalecik Karası and Merlot wines. When the results were compared on the basis of geographical area, Aegean red wines were found to contain generally higher levels of biologically important phenolics and thus to have more antioxidant capacity compared to the wines of the other regions studied.

## Introduction

Phenolic compounds in wine are important because they contribute to the color, taste and body of the wine. The skin and seeds of the grape berry are rich in phenolic compounds. In addition to ethanol, red wine contains a wide range of polyphenols derived from the skin of the grapes, with important biological activities [1]. The grape varieties, climate, soil, agronomical techniques used, the health of grapes and the wine making processes are the most important factors on the phenolic content of red wine [2]. According to those factors, red wine contains many important antioxidant phenolics at different level such as the flavanols quercetin and myristein, (10-20 mg/L), the flavanols catechin and epi(gallo)catechin (up to 270 mg/L), gallic acid (95 mg/L), condensed tannins (catechin and epicatechin polymers (2 500 mg/L) and also polymeric anthocyanidins [3]. Red wine diluted 1,000-fold has been shown to inhibit the *in vitro* oxidation of human LDL (low density lipoproteins) significantly more than α-tocopherol [4]. 

The moderate consumption of red wine has a relatively great benefit in the prevention of atherosclerosis and coronary heart disease (CDH). Regular consumption of red wine has been hypothesized to be the most likely cause for this phenomenon known as the “French Paradox” [5,6,7]. 

Polyphenolic compounds have been shown to possess different biological properties, such as anti-inflammatory responses, prevention of low density lipoprotein oxidation, antihypertensive and antithrombic effects, and antiviral and carcinostatic properties [8,9].

Catechin and epicatechin are epimers, with (-)-epicatechin and (+)-catechin being the most common optical isomers found in nature and the predominant flavanols in red wine. Many studies on the health benefits studies of red wine have been linked to the catechin content. Catechins possess antioxidant properties [10,11] and exert a more potent antioxidant effect than flavonols and polymeric anthocyanidins [12,13]. (+)-Catechin is effective in blocking the growth of the human cell lines originating from cancers of the prostate and breast [11]. During vinification, only a portion of catechins and procyanidins is extracted from seeds and is diffused to the wine [14]. Epicatechin is reported to have insulin mimetic action with protective effects on erythrocytes in a manner similar to insulin and also offers antioxidant protection against lipid peroxidation and inhibits platelet aggregation [10]. 

Gallic acid (3,4,5-trihydroxybenzoic acid) is found in different plants. The main sources of gallic acid in wine are grape seeds and oak cooperage. Gallic acid is also present in grape stems and can be increased by whole cluster fermentations [15]. Gallic acid concentrations of wine range from 10 to more than 100 mg/L and is important in inhibiting cell proliferation and inducing apoptopis of LNCaP cells [16]. Gallic acid is also used as an antioxidant food additive, e.g, lauryl gallate and other alkyl esters, agents that demonstrate the ability to kill animal tumor cells by inducing apoptosis [17] and exert a beneficial effect in several pathobiologies such as CAD, thrombosis, and cancer after moderate consumption of wine [18]. 

The phenolic acids such as caffeic acid, syringic acid, sinapic acid, protocatechuic acid, ferulic acid and 3,4-dihydroxyphenylacetic acid (PAA) exert a direct antiproliferative action on the human breast cancer T47D cell line, at concentrations more or less similar to those expected from normal consumption of foods [18]. Vanillic acid is the oxidized form of vanillin. The chemical designation is 4-hydroxy-3-methoxybenzoic acid and used as a central nervous system stimulant, respiratory stimulant, and analeptic.

**Figure 1:**
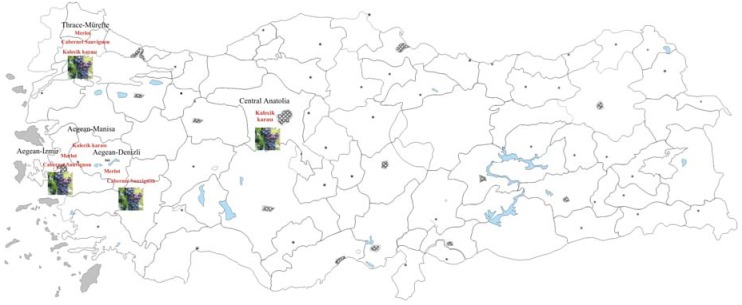
The main wine regions of Turkey.

Turkey, with 535,000 hectares of vineyard area, ranks fourth worldwide after Spain, France and Italy. However, only 3-4 % of grapes are still used for wine production. In Turkey, grape varieties of local and French origin are used for wine production [19]. Figure 1 shows the main wine regions of Turkey.

The aim of this research was to study the representative and characteristic varietal red wines from the four main wine regions of Turkey, by determining their biologically important antioxidant phenolic compounds and their antioxidant capacities to evaluate their potential antioxidant abilities. 

## Results and Discussion

**Figure 2:**
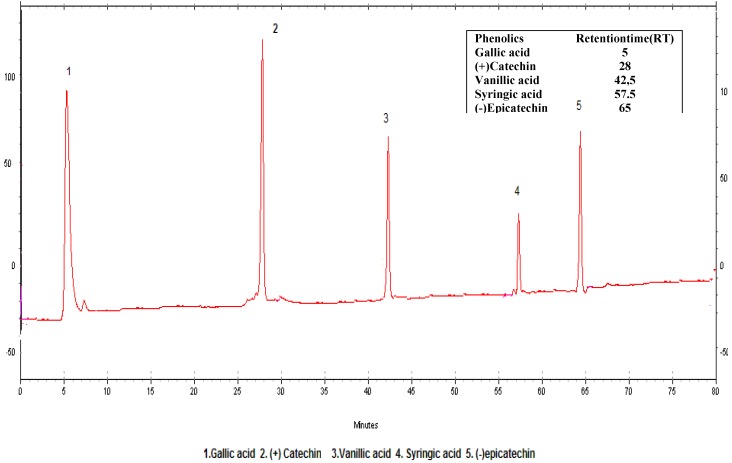
HPLC chromatogram of the phenolic standards.

**Figure 3:**
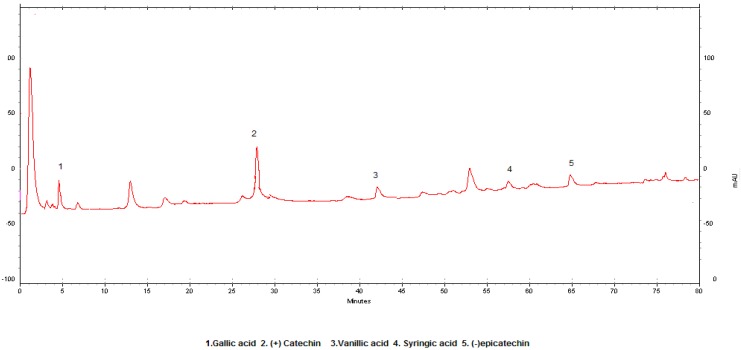
Sample HPLC chromatogram of the Kalecik karası wine from Central Anatolia (Kalecik).

The HPLC conditions employed are critical for both the separation and detection of the five antioxidant phenolics of the red wines analyzed. Table 1 shows the LOD, LOQ and R^2^ values, Figure 2 the standard HPLC chromatogram and Figure 3 the representative HPLC chromatogram of Kalecik Karası wine from Ankara region. 

The R^2^ values range from 0.9987 to 0.9999. These values show the good repeatability and linearity of the chromatographic analysis (Table 1).

**Table 1:** LOD, LOQ and R^2^ values of the determined phenolic compounds.

Compound	LOD (mg/L)	LOQ(mg/L)	A	b	R^2^	RSD
1. Gallic acid	0.025	0,078	64.021	-5800	0.9996	1.2
2.(+)-Catechin	0.075	0,200	31.572	765.06	0.9987	1.1
3.Vanilic acid	0.050	0,150	68.958	4807.2	0.9999	3.7
4.Syringic acid	0.010	0,035	93.157	2492.6	0.9997	3.2
5.(-)Epicatechin	0.095	0,285	24.128	768.80	0.9998	0.8

LOD: Limit of detection; LOQ: Limit of quantification

The results show that gallic acid, (+) catechin and (-) epicatechin are the major phenolic antioxidant substances of the Turkish red wines, ranging from 40.8. to 70 mg/L for gallic acid, 19.3 to 33.9 for (+) catechin and 26.6 to 61.8 for (-) epicatechin (Table 2). Comparing the total phenol and antioxidant activities of the Turkish red wines, Cabernet Sauvignon wines show higher values of total phenol and antioxidant capacities than Merlot and Kalecik Karası wines. 

**Table 2:** Phenolic compounds of Turkish Red wines from differents wine regions (mg/L).

Variety	Origin of Region	n	Gallic acid	(+)- Catechin	Vanilic acid	Syringic acid	(-)- Epicatechin
Kalecik karası	Central Anatolia	3	40.85 ± 0.5452 c	31.98 ±0.148 a	4.66 ±0.0525 a	4.39 ± 0.0225 c	26.61 ± 0.1050 b
Kalecik karası	Thrace-Mürefte	3	45.48 ± 0.1380 c	33.98 ±0.148 a	5.22 ± 0.110 a	4.71 ± 0.095 b	29.48 ± 0.3403 b
Kalecik karası	Aegean-Denizli	3	42.71± 0.4430 c	32.78 ± 0.143a	4.99 ± 0.0329a	4.52 ± 0.074 b	27.98 ± 0.3402 b
Merlot	Thrace-Mürefte	3	52.12 ± 0.023 b	19.38 ± 0,04 c	4.85 ± 0.0410 a	4.82 ± 0.020 b	27.10 ± 0.210 b
Merlot	Aegean-İzmir	3	52.11 ± 0.021 b	20.29 ± 0.122 c	4.66 ± 0.051 b	5.09 ± 0.0825 a	26.37 ± 0.0850 c
Merlot	Aegean-Manisa	3	51.99 ± 0.020b	19.53 ± 0.122c	4.73 ± 0.0410 b	4.93 ± 0.0713b	26.98 ± 0.0671 c
Cabernet Sauvignon	Thrace-Mürefte	3	61.22 ± 1.090 a	25.730 ± 0.600 b	4.77 ±0.0500 a	5.12 ± 0.0125 a	51.91 ± 0.418 a
Cabernet Sauvignon	Central Anatolia	3	61.56 ±0.187a	20.23 ± 0.135c	4.97 ± 0.0432a	5.21 ± 0.0234a	50.66 ± 0.387a
Cabernet Sauvignon	Aegean-Denizli	3	68.430±0.05a	24.351±0.130 b	4.99 ±0.0275 a	5.012±0.148 a	58.93 ± 0.0250 a

*The differences defined with different letters between the two means are statistically significant (p < 0.05)

The varietal wines from the İzmir region were found to contain significantly higher amounts of phenolic antioxidants, thus to have higher amounts of total phenol and higher antioxidant capacity compared to the wines of the three grape varieties from other regions for all of the three grape varieties. Apart from the grape cultivar, this could be mainly due to the different environmental and growing factors of the regions [20]. On the other hand, the maceration techniques also play a role in the antioxidant capacity of red wines. According to research on this subject, the antioxidant activity of red wines exclusively from Syrah and Grenache varieties from the Rhône Valley region made with long maceration was found to have a higher capacity and to be responsible for 60% of the relative inhibition of LDL oxidation in the human body [20]. In our research Central Anatolian wines and Aegean wines contained a significantly higher amount of these antioxidant rich phenolics, as shown in Table 3.

The results show that the red wines from the South Aegean (İzmir) region generally present higher amounts of total phenol and antioxidant phenolics, thus have a higher antioxidant capacity (AC) compared to the wines of other regions. The concentration of total phenol as determined by the Folin±Ciocalteu method varied from 1,480 to 2,410 mg/L GAE. Cabernet Sauvignon wines are significantly richer in total phenol and show high levels of AC (18.1-22.6) among the varietal Turkish red wines. These values are slightly higher than those of French Cabernet Sauvignon wines but lower than those of Pinot Noir wines [4]. Native Anatolian variety Kalecik Karası shows a small amount of total phenol compared to the other varieties. The phenolic content and antioxidant capacities of Turkish red wines seem to be comparable with those of the wines from Spain, Portugal and California [21]. However, it doesn’t seem to be plausible to claim a direct relation between AC and total phenol content of the varieties.

**Table 3:** Antioxidant capacities and total phenol content of the Turkish red wines.

Variety	Region	ACª (mmol/L)	Total phenol (mg/L GAE)
Kalecik karası	Central Anatolia	15.8	1070
Kalecik karası	Thrace-Mürefte	16.3	1130
Kalecik karası	Aegean-Denizli	18.7	1420
Merlot	Thrace-Mürefte	15.8	1510
Merlot	Aegean-İzmir	17.6	1480
Merlot	Aegean-Manisa	17.1	1720
Cabernet Sauvignon	Thrace-Mürefte	18.1	2320
Cabernet Sauvignon	Aegean-İzmir	22.6	2410
Cabernet Sauvignon	Aegean-Denizli	19.3	2390

ªAntioxidant capacity; b Expressed in mg Gallic Acid Equivalent/L (mg GAE/L); ** The values are the avarage of the two replicates.

## Experimental

### Wine samples

Nine different varietal Turkish wines, produced from three different varieties (Kalecik Karası, Merlot and Cabernet Sauvignon), originating from four different wine regions of Turkey (Central Anatolia/Ankara, Thrace/Mürefte, West Aegean/Denizli and South Aegean/İzmir) were taken after production from the wineries. All the grapes were harvested from the proper vineyards of wineries and wine processes were conducted under our control to ensure a similarity of samples from each variety and each region. In each case, classic maceration processes were applied at 26-28 ºC in stainless steel tanks and immediately after fermentation the wines were transferred directly to the oak barrels .

The wineries from the geographical area are: -Kavaklıdere Wineries S.A (Ankara-Central Anatolia), Sevilen Wineries S.A (İzmir- South Aegean), Aral Wineries S.A. (Mürefte-Thrace), Pamukkale Wineries S.A.(Denizli-East Aegean). 

Kalecik Karasi, accepted as the most important red grape variety of Turkey, is a native Turkish grape cultivar originally from Ankara/Kalecik (Central Anatolia). Merlot and Cabernet Sauvignon are French origin grape cultivars produced in different wine regions of Turkey. The samples were taken from the wineries before bottling, after the maturation in French oak barrels during the six months following the 2006 harvest. All wine samples were stored at 4 ºC in glass vials under an argon atmosphere and protected by foil against sunlight-induced isomerization during storage and sample handling. Analyses were completed within a month after sampling.

### Antioxidant capacities

Antioxidant capacity was determined by the total antioxidant method of Randox using a Randox kit (catalog no. NX2332, Randox Laboratories Ltd., Cremlin, U.K). The assay is based on the 2,2’-azinodi (3-ethylbenzthiazoline sulfonate, ATBS) incubated with a peroxidase (metmyoglobin) and H_2_O_2_ to produce the ABTS^+^ radical cation. This technique measures the relative ability of antioxidant substances to scavenge the ABTS radical cation (ABTS^+^) generated in the aqueous phase, compared with standard amounts of the synthetic antioxidant Trolox^®^ (6-hydroxy-2,5,8-tetramethyl chroman-2-carboxylic acid), a water-soluble vitamin E analogue. The basic principle is the reduction of the blue-green ABTS^+^ radical by electron- or hydrogen-donating antioxidants, which is measured by suppression of its characteristic long wave absorption spectrum. ABTS^+^ is generated through the peroxidatic action of metmyoglobin in the presence of ABTS, using metmyoglobin (2.5 ~tM), H_2_O_2_ (75 p.M) and ABTS (150 ~M) (final concentrations). The assay was made up with 980 μL of ABTS^+^ solutions and 20 μL of the sample (at a dilution of 1:50 in water). This has a relatively stable blue-green color, which is measured at 600 nm. Antioxidant in the added sample causes the suppression of this color production to a degree proportional to their concentration [4]. This analytical procedure has been applied to physiological antioxidant compounds in radical scavenging drugs, and an antioxidant ranking based on their reactivity relative to a 1.0 mmol/L Trolox^®^ standard has been established. The Trolox equivalent antioxidant capacity of plasma from an adult references population has been measured and the method optimized and validated [22,4].

### Total Phenolics

Total phenolic contents of the wine samples were determined spectrophotometrically according to the Folin-Ciocalteu colorimetric method [23], calibrating against gallic acid standards and expressing the results as mg gallic acid equivalents (GAE) extract. Data presented are average of three measurements.

### Standards and Reagents

The following substances were purchased from Sigma (St Louis, MO) and used for calibration: (+) catechin, (–)catechin, syringic acid, gallic acid and vanilic acid. Stock solutions of all the standards (1,000 mg/L) were prepared in water-acetic acid-acetonitrile (62:6:32 v/v/v). Working standards were made by diluting the stock solution in the same solvent. Both stock and working standards were stored at -18 ºC until further use [24]. All wine samples were prepared in water-acetic acid-acetonitrile (62:6:32 v/v/v) by diluting at a ratio of 1:2. 

### HPLC analysis

A Thermo Finnigan instrument equipped with a Surveyor Photodiode Array Detector (PDA), Surveyor auto sampler, Surveyor LC Pump (Quaternary gradient) and Chrome Quest Chromatography Workstation were used. The column was a Wakosil II 5 C18RS, 5 µm (250x4.6 mm I.D.). A Rheodyne injection system (7725i) with a 25 μL loop was used. The phenolics in the samples were detected by scanning at 250-500 nm wavelengths with PDA detector. The monitoring wavelength was 278 nm. Gradient elution of three solvent were used: Solution A: acetic acid-water (1/99 v/v); Solution B: acetic acid -water (6/94 v/v); Solution C: acetic acid-acetonitrile-water (5/30/65 v/v/v). The following gradients were used: % 100 A 0-15 min, % 100 B 15-30 min, % 90 B-%10 C 30-50 min, % 80 B-% 20 C 50-60 min. Flow rate: 0.5 ml/min, The temperature of column: 22.5 ^o^C

### Statistical analysis

Statistical analysis results were obtained by using one-way ANOVA [25]. Because of each variety is not found in each region, the variety and geographical area have been chosen as a two-difference factor and one-way anova has been applied for the variety and for the geographical area separately. 
